# Is sexism associated with the use of relational aggression by young adults? The moderating effect of gender

**DOI:** 10.1177/02654075261430507

**Published:** 2026-02-28

**Authors:** Marion Chatelois, Stéphanie Boutin, Alexa Martin-Storey, Michèle Déry, Mélanie Lapalme

**Affiliations:** 1 University of Quebec in Montreal, Canada; 2 University of Sherbrooke, Canada

**Keywords:** proactive relational aggression, reactive relational aggression, hostile sexism, benevolent sexism, young adults

## Abstract

Relational aggression refers to behaviors intended to harm a target’s social relationships either in response to a threat (reactive form) or with an ulterior motive (proactive form). The use of relational aggression is associated with internalizing symptoms such as depression, anxiety, and somatic complaints, as well as externalizing problems including conduct disorder symptoms, attention problems and oppositional defiant disorder symptoms (Murray-Close et al., 2016). Considering these consequences, the factors that may explain the use of relational aggression need to be explored. By adopting a macro-systemic perspective, this paper addresses the role that sexism may play in the use of relational aggression by young adults. Using data from an ongoing longitudinal study of social adaptation during the transition into adulthood (571 participants aged 17–22 years; 49.3 % women), this paper focused on the association between relational aggression and sexism in young adults, and on the moderating role of gender in this association. Results showed a positive association between hostile sexism and proactive as well as reactive relational aggression. However, benevolent sexism was associated with neither form of relational aggression. Gender only moderated the association between hostile sexism and reactive relational aggression, which was stronger for men than for women. These results allow for a more complex analysis of relational aggression and show the relevance of variables related to the broader social context, such as sexism, for understanding young adults’ aggressive behaviors.

## Abstract (fr)

L’agression relationnelle désigne des comportements qui visent à nuire aux relations interpersonnelles d’une personne et qui peuvent être perpétrés en réponse à une menace (forme réactive) ou avec un motif ultérieur en tête (forme proactive). La perpétration d’agression relationnelle est associée à des symptômes intériorisés comme la dépression, l’anxiété et les plaintes somatiques, ainsi qu’à des problèmes extériorisés comme des symptômes de troubles de conduite, des problèmes d’attention et des symptômes de trouble oppositionnel (Murray-Close et al., 2016). Considérant ces conséquences, les facteurs qui peuvent expliquer le recours à l’agression relationnelle doivent être explorés. Par l’adoption d’une perspective macro-systémique, cette étude s’intéresse au rôle potentiel que pourrait jouer le sexisme dans l’utilisation de l’agression relationnelle par les jeunes adultes. Utilisant des données secondaires issues d’une étude longitudinale portant sur l’adaptation sociale durant la transition vers l’âge adulte (571 participants âgés de 17–22 ans; 49,3% femmes), cette étude se penche sur l’association entre l’agression relationnelle et le sexisme chez les jeunes adultes, et sur le rôle modérateur du genre dans cette association. Les résultats montrent une association positive entre le sexisme hostile et l’agression relationnelle proactive ainsi que réactive. Cependant, le sexisme bienveillant n’était associé à aucune fonction d’agression relationnelle. Le genre modère seulement l’association entre le sexisme hostile et l’agression relationnelle réactive, qui est plus forte chez les hommes que chez les femmes. Ces résultats permettent une analyse plus complexe de l’agression relationnelle et soulignent la pertinence de s’intéresser à des variables reliées au contexte social plus large, comme le sexisme, pour mieux comprendre les comportements agressifs des jeunes adultes. *When multiple-language abstracts are provided, the authors confirm-- under their own responsibility-- that the translation of the abstract into their mother-tongue language corresponds to the English version and that the translation of the abstract is factually correct and linguistically accurate.

Relational aggression is a form of aggressive behavior where the goal is to hurt another person by using interpersonal relationships to cause harm, usually with the intent of damaging those relationships or the victim’s sense of social inclusion (Murray-Close et al., 2016). In addition to harm done to victims (Kawabata et al., 2020; McField et al., 2023; Prinstein et al., 2001), individuals who report engaging in higher levels of relational aggression report more internalizing and externalizing problems, risky behaviors such as substance use, and symptoms of antisocial, borderline and narcissistic personality disorders (Murray-Close et al., 2016). Using relational aggression may also increase the risk of experiencing social difficulties, as aggressors’ behaviours may be perceived as unpleasant by their peers (Murray-Close et al., 2016).

When studying the correlates of aggressive behaviors, macro-system variables have often been neglected in favor of micro-system variables, such as school, family, or peers (Carrera-Fernández et al., 2021). This approach fails to consider how the norms, beliefs and expectations regarding power and identity could influence young people’s behaviors towards each other. More specifically, norms around gender can affect young people’s perception and use of relational aggression. Women are socially perceived as more gossipy, and relational aggression is often perceived as a stereotypically feminine aggressive pattern, although studies show little gender differences in the frequency of these behaviors (Card et al., 2008; Coyne & Archer, 2004; Eckhaus & Ben-Hador, 2019). Conversely, women’s perpetration of relational aggression is perceived as less acceptable or justified than men’s perpetration (Basow et al., 2007; Coyne et al., 2008). Nevertheless, both young women and young men are concerned about protecting their reputation and fulfilling feminine or masculine norms, especially in honor cultures (Osterman & Brown, 2011). Such concerns are thought to precipitate the use of aggression, as a way to defend one’s standing (Foster et al., 2022). Aggressive behaviors such as relational aggression can then be used to police gender identity and to ensure conformity to heterosexual norms, thereby distinguishing between the majority group and the minorities who challenge hegemonic masculinity and femininity (Carrera-Fernández et al., 2021). For instance, girls use sexualized rumor spreading, slut-shaming, and homophobic labelling to negotiate gendered sexuality and make claims about their own femininity (Miller, 2016). Similarly, homophobic language and homophobic bullying are part of boys’ gender socialization into normative masculinity (Pascoe, 2005, 2013). The role of variables related to social rules and discourses about gender have therefore been underestimated in the understanding of young people’s use of relational aggression. This study takes a broader perspective by examining the association between relational aggression and sexist beliefs in young adults to help better understand the use of relational aggression and prevent the associated consequences.

## Relational aggression

Relational aggression describes behaviors that intend to hurt the other by attacking their friendships and feelings of inclusion (Crick & Grotpeter, 1995). Relational aggression can have different functions which are derived from distinct theoretical underpinnings. On one hand, proactive relational aggression occurs when the perpetrator has an ulterior motive, and it serves a goal-oriented end (Murray-Close et al., 2010, 2016). Acting cold with a peer until they surrender to a demand or threatening to share private secrets to get someone to comply to a wish are examples of proactive relational aggression. Proactive aggression is based on Bandura’s social learning theory (1973), which states that aggression is an acquired behavior influenced by anticipated reward, the instigating factor being the expected success of the behaviour (Kempes et al., 2005). On the other hand, reactive relational aggression typically occurs out of anger, in response to a perceived threat (Murray-Close et al., 2010, 2016). For example, someone might try to publicly ridicule a peer who made them mad or gossip about someone out of anger. According to the frustration-aggression hypothesis (Berkowitz, 1962), reactive aggression is a hostile reaction to a perceived frustration, accompanied by high autonomic arousal (Kempes et al., 2005). It is more closely linked to poor emotional regulation and poor psychosocial adjustment (Card & Little, 2006). In both cases, relational aggression allows aggressors to influence social relationships and ensure dominance or popularity, while suffering few consequences from peers or adults, since relational aggression is perceived as benign or even normative compared to other forms such as physical aggression (Archer & Coyne, 2005; Swit, 2021).

### Relational aggression in young adults

Effective use of relational aggression may require advanced socio-cognitive and verbal abilities (Murray-Close et al., 2016) that are mostly mastered in late childhood and adolescence, during which youth develop stronger peer relationships. Although there seems to be a general decline in the use of relational aggression in late adolescence, there is significant variability in these trajectories, and relational aggression remains prevalent into early adulthood (Coyne et al., 2020; Nelson et al., 2008; Vaillancourt & Farrell, 2021). Indeed, emerging adults are faced with “new rules for appropriate behaviors, new peers to interact with, new social status hierarchies to navigate, and new developmental milestones to manage” (Casas & Bower, 2018, p. 38). The multiplication of social contexts and the increase in autonomy leads to a diversification of emerging adults’ social interactions. This can allow for the continuation or even emergence of relationally aggressive behaviors, as forming new relationships involves negotiating new social hierarchies and reorganizing existing relationships (Casas & Bower, 2018).

However, emerging adults who still rely on relationally aggressive strategies seem to experience a growing array of negative socioemotional consequences (Dahlen et al., 2013; Ostrov & Houston, 2008; Storch et al., 2005). This could be due to accumulating discord in their relationships within a wider range of social contexts that are less tolerant of these tactics, deemed immature and inappropriate (Casas & Bower, 2018). The apparent harmful outcomes of relying on these strategies in early adulthood underscore the importance of investigating the potential variables associated with the use of relational aggression by emerging adults.

### Relational aggression and gender

Although results on gender differences in the use of relational aggression are mixed (Murray-Close et al., 2016), Card and colleagues’ meta-analysis (2008) shows that aggressive boys seem to employ both physical and relational forms of aggression, while girls prefer relational rather than physical forms. These differences could in part be attributed to the way children are socialized (Bussey & Bandura, 1999; Ellemers, 2018). Girls are often discouraged by adults to use physical forms of aggression, and peers tend to criticize behaviors that are inconsistent with gender roles (Kolbert et al., 2010; Maccoby, 1998). Girls may be more likely to use relational aggression because it aligns with typically feminine social patterns (importance placed on intimate relationships, being passive and emotional; Boutin et al., 2017; Carrera-Fernández et al., 2013; Murray-Close et al., 2016). On the other hand, boys might use more direct aggression since being tough, aggressive, and assertive are valued features of the men gender role. However, in young adulthood, men and women do not seem to differ in their overall use of relational aggression (Murray-Close et al., 2010).

Gender stereotypes that lead socializing agents (parents, peers, school) to treat girls and boys differently are internalized by children themselves. These gendered norms can also lead to the development of sexist beliefs and influence the way peers treat each other throughout young adulthood.

## Hostile sexism and benevolent sexism

Sexism is typically understood as a form of prejudice and antipathy toward women (Glick & Fiske, 1996). However, Glick and Fiske (1996) argue that sexism is a multidimensional concept marked by ambivalence and encompassing both a benevolent and a hostile form. This “ambivalent” sexism stems from the patriarchal system that is prevalent in most human societies. Patriarchy describes the power relations between men and women, focusing on men’s control over most economic, legal and political institutions, both in public and private spheres (Glick & Fiske, 1996; Sultana, 2010). The ambivalence within sexism comes from the fact that although women have historically been relegated to domestic roles in this patriarchal system, they still hold dyadic power, such that men generally depend on women for sexual reproduction and the satisfaction of sexual and intimacy needs (Glick & Fiske, 1996). This dyadic power is reflected in the protective attitudes toward women and the admiration for traditional feminine roles (mother, housewife, romantic object) that characterize benevolent sexism. Conversely, hostile sexism holds a more negative tone and portrays women as a subordinate group, whose characteristics justify the social control exerted over them by men (Carrera-Fernández et al., 2013). This form of sexism is often directed toward certain specific groups of women who challenge or threaten men’s dominance and their paternalistic needs (e.g., feminists, powerful women in business or politics; Glick & Fiske, 1996; Lee et al., 2010). Thus, sexism is not simply hostility toward women but may also involve a belief that it is necessary to protect certain types of women and their traditional roles, while still perceiving them in a restrictive and stereotyped way.

Men report higher levels of sexism than women (King et al., 2019; Prather et al., 2012), especially in its hostile form (Carrera-Fernández et al., 2013). Nevertheless, women can also internalize sexist beliefs, and some studies have found no gender differences in benevolent sexism (Carrera-Fernández et al., 2013; Hideg & Ferris, 2016). Indeed, benevolent sexism’s belief that women should be protected and cherished can disarm women’s resistance to hostile ideologies towards their own gender (Glick & Fiske, 1996). Women can also endorse hostile sexist attitudes, which they generally direct towards norm-deviant women who do not match their traditional role conceptions, rather than towards their own in-group (Becker, 2010).

### Developmental pattern of sexism

Children learn gender-specific information growing-up, i.e. the behaviors, roles, attributes and personality traits typically expected of men and women. This gendered socialization is shaped by the interaction of the multiple contexts in which they develop: parents, school, peer groups, mass media, all embedded within a broader culture built around heteronormative patriarchy (Brown et al., 2020). Therefore, early in their development, children can internalize patriarchal gender stereotypes that can lead to sexist beliefs, such as viewing boys as tough and dominant and girls as sweet and fragile, or perceiving men as occupying high-status roles and women as caregivers (Leaper, 2024).

Over time, endorsement of ambivalent sexist attitudes generally follows a U-shaped trajectory: relatively high in early adulthood, lower through middle adulthood, and higher later in life (Hammond et al., 2018). Only men’s benevolent sexism seems to follow a positive linear trajectory across age, possibly because it is less detrimental to relationship goals than hostile sexism (Hammond et al., 2018). Therefore, sexist attitudes are still present in early adulthood, hence the relevance of examining how young adults’ aggressive behaviors could be influenced by how much they still adhere to these beliefs.

## Sexism and relational aggression

Sexism perpetuates the idea that men and women should be treated differently and occupy distinct roles, helping to justify and maintain men’s dominance over the social hierarchy (Ellemers, 2018; Glick & Fiske, 1996). In a similar way, relational aggression can be seen as a way for young adults to gain high social status among their peers, establishing a social hierarchy that allows them to maintain dominance (Murray-Close et al., 2016). People who favor hierarchical over equal intergroup relations can be considered more oriented towards social dominance (Pratto et al., 1994), which has been positively associated with hostile and benevolent sexism (Austin & Jackson, 2019; Christopher & Mull, 2006; Lee, 2013; Rollero et al., 2021; Sibley & Becker, 2012; Sibley et al., 2007) as well as with the use of relational aggression (Gumpel & Gotdiner, 2022; Mayeux, 2014). Moreover, sexism divides people, especially women, into polarized subgroups depending on their ability to adhere to gendered norms and roles, and rewards or punishes them accordingly (Agut et al., 2023; Hammond et al., 2018). Women who are warm, docile and adopt traditional roles (e.g., homemakers) are revered, whereas women who adopt non-traditional roles are seen as threatening and deserving of hostility (Agut et al., 2023). In this gendered hierarchy, individuals who are invested in maintaining sexist norms could be more likely to be aggressive towards people who do not fit these gendered norms, or who threaten their masculinity or femininity. Relational aggression could appear as the ideal form of aggressive behavior to reestablish the gender hierarchy and protect one’s standing, since compared to other forms, it allows for social benefices with little retribution (Archer & Coyne, 2005). Additionally, the gendered hierarchy favored by sexist individuals aims to disadvantage women (Glick & Rudman, 2010). It is thus possible that people who hold sexist beliefs would be more likely to attack something that is particularly important to women: interpersonal relationships (Murray-Close et al., 2016). In short, people who adhere to sexist beliefs are invested in maintaining a specific dominant-dominated gendered hierarchy, in maintaining their own hegemonic masculinity or femininity, and in policing people who do not conform to sexist norms. They may therefore resort to using relational aggression to navigate interpersonal hierarchies and preserve their own place in it.

## Prior works on sexism and aggression

Some existing research has explored the link between sexism or gendered beliefs and bullying or other conduct problems. Two studies have found a positive association between hostile sexism and bullying or positive attitudes toward bullying in teenagers (Carrera-Fernandez et al., 2013, 2021), but these results are contradicted by DeSouza and Ribeiro (2005), who did not find any significant association. The authors explain this lack of association both by the low internal reliability coefficient for hostile sexism in their study, and by the fact that in general, bullying is primarily directed at peers of the same sex rather than peers of the opposite sex, suggesting that sexism might be a better predictor of bullying toward other-sex peers. Regarding benevolent sexism, there seems to be a negative association with bullying (Carrera-Fernandez et al., 2013, 2021). The paternalistic and affective tone of benevolent sexism, in contrast with the harshness of hostile sexism, may explain its seemingly protective role against bullying behaviors (Carrera-Fernández et al., 2021). Additionally, some studies found that teenagers with more traditional attitudes about gender roles have higher levels of misbehavior in school (Heyder et al., 2021), and in boys’ case, more aggressive behaviors (Daff et al., 2020). On the contrary, teenagers with more egalitarian attitudes have fewer conduct problems and better prosocial behavior, especially for boys (King et al., 2019). Finally, Foster and colleagues (2022) have found that honor-endorsing women use reactive relational aggression in response to reputation threats centered around sexual purity, chastity, and fidelity. Similarly to sexist beliefs, honor beliefs imply an expectation to fulfill specific feminine or masculine norms to maintain reputation, and such beliefs are thought to precipitate violence (Foster et al., 2022). Reactive relational aggression could therefore be used to protect hegemonic femininity or masculinity.

Taken together, these findings indicate that sexist attitudes, specifically in their hostile form, can be associated with more aggressive and disruptive behaviors, especially for boys. It is therefore plausible that hostile sexism would be positively associated with relational aggression, especially reactive relational aggression. Indeed, sexist attitudes are deeply ingrained, and individuals can participate in perpetuating these dichotomous beliefs without conscious awareness (Jun, 2018). Hostile sexist beliefs might therefore be particularly activated in response to an affront, leading to a stronger association with reactive relational aggression, an angry reaction that could reflect internalized hostility towards people who do not fit sexist norms or who threaten one’s masculinity or femininity. On the contrary, benevolent sexism might be negatively associated with the use of both reactive and proactive relational aggression, because of its subjectively favorable and protective undertone.

## The present study

Aggressive behaviors have often been studied in terms of individual psychological or biological factors (Carrera-Fernández et al., 2021; Murray-Close et al., 2016). Clarifying the association between hostile and benevolent sexism and relational aggression functions would allow for a broader analysis of the way societal correlates within the macro-system can shape aggressive behaviors between peers. This study will focus on a sample of young adults, since this is a developmental period characterized by a proliferation of social challenges that can be associated with the persistence of relationally aggressive behaviors and sexist attitudes. Although research suggests the existence of a link between sexism, especially in its hostile form, and conduct problems at large, the specific associations with relational aggression are inconsistent and would benefit from further clarifications. Moreover, reactive and proactive aggression have been shown to be characterized by different causal mechanisms, correlates, outcomes, and indications for different types of intervention (Merk et al., 2005). Specifying the distinct associations with both functions of relational aggression could contribute to a better understanding of how to intervene and prevent aggressive behaviors in young adults.

This study has two objectives: (1) to examine if reactive and proactive relational aggression are linked with hostile and benevolent sexism among young adults; (2) to examine the moderating effect of gender on the associations between reactive and proactive relational aggression and both forms of sexism.

## Method

### Participants

This project uses secondary data from a large ongoing longitudinal study on the social adaptation of children with conduct problems. Between 2008 and 2010, 744 students with and without conduct problems (46.8% girls; aged 6–9 years) were recruited in 155 elementary schools from eight French speaking school boards located in four regions of Quebec (Montreal, Montérégie, Estrie and Quebec City).

To recruit a large number of participants (particularly girls) with conduct problems before age 10, two strategies were used. First, most participants (*n* = 339; 44% girls) were recruited based on whether they were receiving services for conduct problems in public schools. The Achenbach System of Empirically Based Assessment was used to ensure the recruitment of participants with conduct problems (ASEBA; Achenbach & Rescorla, 2001). Parent and teacher completed the DSM-oriented scales for oppositional problems and conduct problems. To be selected, children had to score at or above the 93rd percentile (i.e., in the at-risk or clinical range for conduct problems) according to either the parent or the teacher’s report. All girls under ten years old and around one out of four randomly selected boys receiving services for conduct problems at school were invited to participate to the ASEBA screening, with a participation rate of 75.1%. There were no differences in grade level, poverty level of the school or in participation rates of boys and girls. More than two-thirds of participants attended a school located in a high poverty neighborhood (Government of Quebec, 2013).

Second, to identify students with conduct problems who might not have been signaled to school professionals by teachers and might therefore not be receiving services, a multi-gated method of systematic classroom-based screening was applied to 881 students in first to third grade attending schools from low-income neighbourhoods. There was a participation rate to the screening of 71.5% with no differences in rates of participation of boys and girls nor in grade level. Once again, the parents and teachers had to complete the oppositional disorder and conduct disorder scales from the ASEBA, and the same threshold was used. Ninety-five participants with conduct problems (57.9% girls) were recruited through this second strategy. Participants without conduct problems were also recruited through these classroom screenings, for comparison purposes. Approximately one out of three students who did not meet the risk threshold on the ASEBA scales was randomly selected. Therefore, in the final sample at study inception (*n* = 744), 434 participants (44.7% girls) had conduct problems and 310 participants (49.7% girls) did not.

The longitudinal project employs a repeated measures design at 12-month intervals. The average annual attrition rate is 1.67%. Data used in the current project is from the 12^th^ yearly data collection (2019–2022) of this longitudinal study since it was the only wave of data in which the variables of interest were measured. Participants (*n* = 571) are aged 17.32–22.02 years (*M* = 19.43; *SD* = 0.96), and 57.1% of them had conduct problems in childhood (at study inception). The sample consists of 49.3% women, 49.5% men, 0.7% nonbinary, and 0.5% other gender identity. When asked which population group they identify with, 89% of participants identify as White, 4.5% as Black, 1.7% as Latino American, 1.6% as Native, 1.2% as Arab, 0.3% as Chinese, 0.2% as Filipino, 0.2% as South East Asian, 0.2% as Occidental Asian, 0.2% as Japanese, and 2.4% identify with another population group (participants could choose more than one group). Regarding sexual orientation, 82.9% of participants are heterosexual, 7.7% are bisexual, 2.3% are gay or lesbian, 2.8% are queer, pansexual or allosexual, 1% are asexual, and 1.4% are uncertain or questioning (0.5% other). Most participants are full-time (46.3%) or part-time (4.5%) students, and many work full-time (31.6%), or part-time (38.3%). Concerning educational status, most participants’ highest educational level is a high school diploma (49.1%). Participants’ median annual income is between C$10,000 and C$14,999. Finally, their family’s median annual income is between C$80,000 and C$89,999.

### Measures

#### Relational aggression

Relational aggression was assessed using Morales and Crick’s Self-Report of Aggression and Social Behavior Measure (SRASBM; unpublished measure, 1998)^1^. The Proactive Relational Peer Aggression scale (5 items; e.g., when I want something from a friend, I’m cold or indifferent to them until I get what I want) and the Reactive Relational Peer Aggression scale (6 items; e.g., I get upset or angry if a friend wants to get close to someone else) were used in this study. Participants answered on a seven-point Likert scale ranging from 1 (not at all true) to 7 (very true). Several studies have reported adequate internal consistency for this scale, with Cronbach’s alpha ranging from .71 to .78 for the different SRASBM scales (e.g., Bailey & Ostrov, 2008; Lento-Zwolinski, 2007). However, in our study, both reactive relational aggression and proactive relational aggression had unsatisfactory internal consistency (*α* = .63 and *α* = .54 respectively). A confirmatory factorial analysis was thus tested to verify the structure of the scales. The model had good fit indices ( $X^{2\;}$ = 61.87, *p* = .02; *CFI* = .97; *TLI* = .95; *RMSEA* = .03 [.01, .04]; *SRMR* = .04) and showed that the items did group into the two intended scales. As such, the use of these latent factors was preferred to the use of observed scales in our model, and this analytical choice is further explained below. Also, a square root transformation was performed on the items to improve normality prior to analysis.

#### Sexism

Hostile and benevolent sexist attitudes were measured using the Inventory of Ambivalent Sexism in Adolescents (IASA) by de Lemus and colleagues (2010).^1^ It contains two 10-item scales that assess hostile (e.g., boys should control who their girlfriend interacts with) and benevolent (e.g., girls should help their mothers at home more than boys) sexist attitudes. A Likert-type scale ranging from 1 (strongly disagree) to 6 (strongly agree) was used, and an average score was calculated for each subscale, with a higher score indicating more sexist attitudes. In this study, both scales had good internal consistency (*α* = .84 for hostile sexism and *α* = .85 for benevolent sexism).

#### Gender

Participants’ gender was determined based on their response to the question, “What gender do you identify with?”. The choices were woman, man, non-binary, and other (with the option to specify for the latter response choice). Participants who identified as a gender minority (i.e., transgender, non-binary or gender fluid) were removed from the analytical sample since their sexist attitudes as conceptualized by the ambivalent sexism theory may be different from cisgender participants and because there were too few participants endorsing these identities to do comparison analyses (Cross et al., 2021). Women were coded 0 and men were coded 1.

### Procedure

Data used for this study was collected via in-person or video conference sessions (due to COVID-19) led by graduate-level research assistants having received a three-day formal training. Participants were presented with a full description of the study and a consent form. They were then invited to answer multiple questionnaires: research assistants read the questions and answer choices out loud and noted down the participant’s responses. Each participant received an C$80 financial compensation for their participation to the 12^th^ wave of data collection. Both the longitudinal study and the current study have been approved by their respective ethics committee.

### Analytical strategies

Missing data was <5% for gender, sexism and relational aggression. Little’s MCAR test for these variables was non-significant, $X^{2}$ (561) = 97.55, *p* = .998, allowing us to infer that data was missing completely at random. Missing data was thus handled by the full information maximum likelihood (FIML) imbedded in Mplus, which was used to produce our analyses (version 8.10; Muthén & Muthén, 1998–2023).

Associations between hostile and benevolent sexism and reactive and proactive relational aggression (objective 1) were estimated using a hybrid structural equation model, in which the predictors were observed variables and the two outcomes were modeled as latent factors. Considering that the use of observed mean scores assumes equal contribution of all items and that the relational aggression scales had low internal consistency in our sample, the use of observed variables would have introduced substantial measurement error. Measurement error can be a problem as it inflates residual variance and reduces the precision of structural estimates. Modeling reactive and proactive aggression as latent factors allowed us to separate true-score variance from item-specific error, allowing items to contribute proportionally to their factor loadings. Doing so, latent factors provide more reliable estimates of the underlying constructs (Rdz-Navarro, 2019). Our confirmatory factor analysis further supported this strategy, with good fit indices and item clustering onto the two intended functions of relational aggression. As for the sexism scales, observed scores were used rather than latent factors to maximize statistical power. Given the abnormal distribution of the relational aggression data, the model was estimated using a robust estimator (maximum likelihood estimation with robust standard errors [MLR]).

To test the influence of gender on the associations between both forms of sexism and both functions of relational aggression (objective 2), two interaction terms with each form of sexism were created and entered in the model. Finally, considering the recruitment strategies and the sample composition, conduct problems at study inception (0 = without conduct problems; 1 = with conduct problems) were controlled for in our model. Interactions terms were also created to test if conduct problems at study inception influenced the association between sexism and relational aggression. The results were non-significant.

## Results

### Descriptive statistics

Correlations and descriptive statistics can be found in Table 1. Results showed significant and positive correlations between both forms of sexism and both functions of relational aggression (*r* ranging from .09 to .26). Benevolent sexism was correlated with gender, so that being a man was associated with higher benevolent sexism. Being a woman was associated with higher reactive relational aggression. Age was not associated with any study variables and was thus not included in the estimated models.

**Table 1.** Correlations and descriptive statistics of study variables.

Variables	1	2	3	4	5	6	7
1. Age	----						
2. Conduct problems	−.01	----					
3. Gender	.00	.03	----				
4. Benevolent sexism	.01	.23**	.36**	----			
5. Hostile sexism	−.02	.22**	−.05	.49**	----		
6. Reactive RA	−.05	.13**	−.10*	.12**	.26**	----	
7. Proactive RA	−.07	.05	−.05	.09*	.19**	.50**	----
*N*	561	561	561	555	555	554	554
Mean	19.44	43.5% with CP	49.7% women	2.03	1.69	0.51	0.24
*SD*	0.96			0.94	0.89	0.61	0.47
[Min-Max]	[17.3–22.0]			[0–4.7]	[0–4.3]	[0–4.8]	[0–3.6]

*Note.* For the correlations and descriptive statistics, a score of reactive RA and a score proactive RA were created for which answers to each item were summed (a higher score indicates higher RA). CP = Conduct Problems.

*p < .05. **p < .01.

### Objective 1: Association between relational aggression and sexism

**Figure 1.**
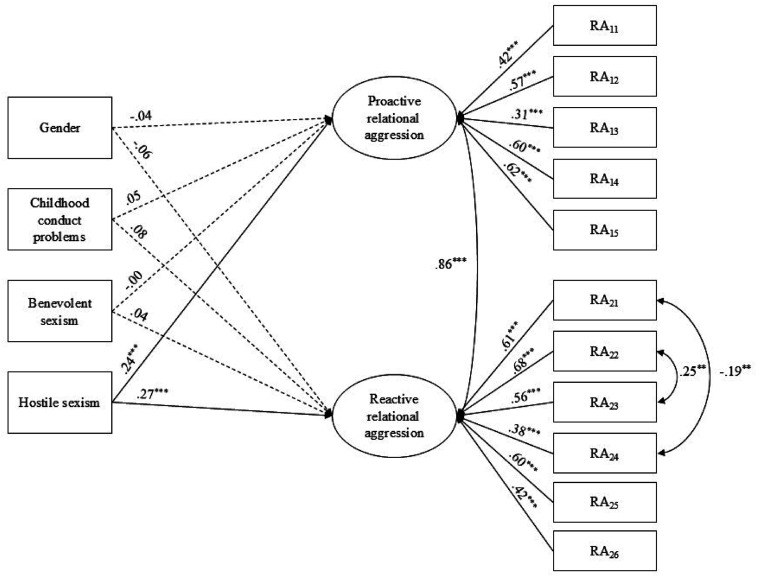
Estimated model (*n* = 554) for the associations between sexism and relational aggression. Entries are standardized coefficients. Dash lines represent non-significant links and full lines represent significant links. $X^{2}$ = 131.09, *df* = 77, *p* < .001; RMSEA [90% CI] = .04 [.03, .05]; SRMR = .04; CFI = 0.93; TLI = 0.91. ^**^p < .01. ^***^p ≤ .001.

The first objective of this study was to examine if there was an association between both functions of relational aggression, hostile sexism, and benevolent sexism. Results show that having more hostile sexist attitudes was related to a greater use of both reactive and proactive relational aggression (see Figure 1). However, benevolent sexism was not associated with either function of relational aggression.

### Objective 2: Moderating effect of gender

**Figure 2.**
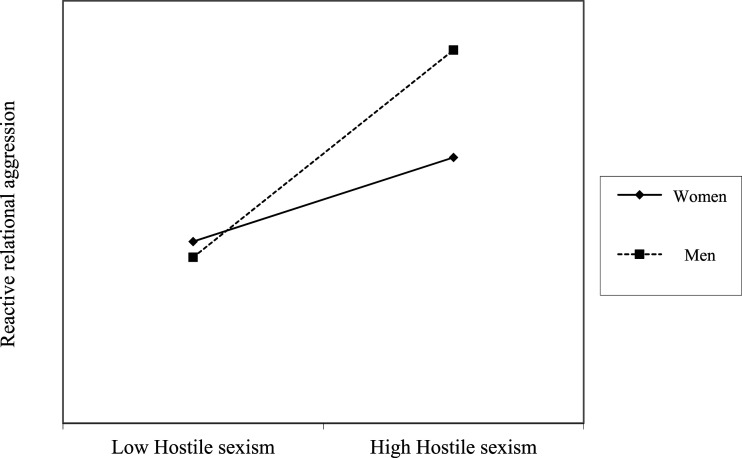
Graphic representation of the moderating role of gender on the association between hostile sexism and reactive relational aggression.

The second objective of this study was to examine the moderation effect of gender on the associations between relational aggression and sexism. Findings from moderation analyses show that gender moderated the association between hostile sexism and reactive relational aggression. The association between hostile sexism and reactive relational aggression was significantly stronger for men than it was for women (see Figure 2). The simple slope of hostile sexism on reactive relational aggression remained significant for men (*b* = .14, *p* < .001) as well as for women (*b* = .06, *p* = .048). Gender did not moderate the association between hostile sexism and proactive relational aggression.

## Discussion

The perpetration of relational aggression is linked to important consequences not only for the victims, but for the perpetrators (internalizing and externalizing symptoms, social difficulties, risky behaviors, etc.). Given these negative outcomes, understanding the mechanisms that influence the use of relational aggression could contribute to preventing it. Focusing on emerging adulthood, this study examined the influence of hostile and benevolent sexist beliefs, a sociocultural factor derived from the patriarchal social discourse through which young people are socialized, on the perpetration of reactive and proactive relational aggression. This study also aimed to examine whether gender moderated the links between these different forms of sexism and both functions of relational aggression. Participants who reported more hostile sexist attitudes were more likely to use both reactive and proactive relational aggression, whereas benevolent sexist attitudes were not associated with either function of relational aggression. Moreover, the association between hostile sexism and reactive relational aggression was moderated by gender, such so that hostile sexism was more strongly associated with the use of reactive relational aggression for men than for women.

### Associations between sexism and relational aggression

Results from structural equation modeling support the hypothesis that in its hostile form, sexism was positively associated with the perpetration of both functions of relational aggression. However, benevolent sexism was associated with neither function of relational aggression in young adults. Although the scientific literature is sparse, there is some consistency between the results obtained in the present study and previous work. Several authors have found a positive association between hostile sexism or traditional attitudes toward gender roles and different aggressive behaviors, such as bullying (Carrera-Fernández et al., 2013, 2021) or relational aggression in a romantic relationship (Prather et al., 2012). Sexism, especially in its hostile form, points to a hierarchical perception of relationships between individuals, where everyone is expected to uphold certain roles based on their gender. Sexist attitudes typically relegate women to a subordinate social position in comparison to men, therefore justifying men’s structural power (Glick & Fiske, 1996). The association between hostile sexism and both functions of relational aggression in the present study supports the idea that an individual who tends to adhere to sexist beliefs is more likely to resort to aggressive behaviors that allow them to control their relationships with their peers and to maintain dominance within the gendered hierarchy they strive to maintain. Hostile sexist beliefs, which are often deeply ingrained and internalized, could lead an individual to have angry and hostile reactions to affronts or threats to their position in the gendered hierarchy (reactive relational aggression), as well as try to manipulate this hierarchy to their advantage in a more instrumental way (proactive relational aggression).

However, results from DeSouza and Ribeiro (2005) contradict the previous findings, as these authors did not find any association between hostile sexism and aggression. This difference could be attributed to the fact that they measured aggression in a broader way, including general physical and verbal aggressive behaviors, rather than focusing on relational aggression. Relational aggression is a more status-oriented behavior that is often associated with an increase in popularity among peers (Casper et al., 2020; Mayeux, 2014). It is then plausible that it would be more strongly linked to hierarchical beliefs such as hostile sexism than other forms of aggression. Relational aggression is also perceived as less serious and represents an alternative aggressive strategy that offers a better cost-benefit balance than other forms of aggression (Archer & Coyne, 2005; Swit, 2021). People who seek to protect their position in a precarious gendered hierarchy might resort to this more socially acceptable form of aggression.

Furthermore, in several studies, benevolent sexism is negatively associated with bullying perpetration (Carrera-Fernández et al., 2021; DeSouza & Ribeiro, 2005) or positive attitudes toward bullying (Carrera-Fernández et al., 2013). As a result, some authors have hypothesized that benevolent sexism may hold a protective effect in relation to individuals’ aggressive behaviors. Benevolent sexism promises protectiveness and affection, especially toward women who fulfill gendered expectations, and it is said to facilitate cooperation by appealing to both men and women (Agadullina et al., 2022). Therefore, people who hold more benevolent sexist attitudes might be less likely to resort to aggressive behaviors, or at least these attitudes may not strongly influence aggression. This could account for the absence of a significant association between benevolent sexism and both functions of relational aggression in this study. Reactive relational aggression implies an antipathic and angry arousal that is contradictory to benevolent sexism’s protective tone. Additionally, benevolent sexism leads to an increased support for the status quo and a perception that the system, or gendered hierarchy, is fair (Jost & Kay, 2005), which might be unreconcilable with the aggressive premeditated goals of proactive relational aggression.

However, it should be noted that the hostile and benevolent forms of ambivalent sexism are still correlated; they are complementary and mutually reinforcing attitudes (Glick & Fiske, 2011). The unsatisfactory internal consistency of the relational aggression measure and the lack of variability in the participants’ responses, specifically in the proactive subscale, could contribute to the absence of association between benevolent sexism and proactive relational aggression. Considering this reliability issue and the fact that benevolent sexism also contributes to maintaining men’s patriarchal power, the association between benevolent sexism and relational aggression, especially proactive aggression, would benefit from further clarifications. For instance, benevolent sexism might not be associated with increased perpetration of proactive or reactive relational aggression, but it could lead to increased support of these aggressive behaviors, since it indirectly justifies violence through victim blaming and failing to recognize violence (Bareket & Fiske, 2023).

### The role of gender

Moderation analyses indicate that hostile sexist attitudes were more strongly associated with the use of reactive relational aggression among men than among women. These results could be attributed to the distinct way in which hostile sexist attitudes present themselves in men and women. It is possible that hostile sexism in women reflect their tendency to embrace or reject cultural norms and expectations, whereas men’s hostile sexism would be primarily related to internal motives, such as a desire for social control over women (Glick & Fiske, 1996). Women may be perceived as competition, threatening men’s dominant position through their sexuality, feminism or ambition, which may elicit antipathy and derogatory behaviors from men (Austin & Jackson, 2019; Bareket & Fiske, 2023; Glick & Fiske, 1996). Men also experience more anxiety over their gender status than women do, since manhood is seen as a precarious social status that needs to be earned and held (see Vandello & Bosson, 2013 for a review about precarious manhood). It seems plausible, then, that men who hold more hostile sexist attitudes would be more likely than women to have aggressive and angry reactions to perceived threatening provocation to their gender status, which may explain the stronger association between hostile sexism and reactive relational aggression among men. Additionally, women’s perpetration of relational aggression is generally perceived more negatively than men’s (Basow et al., 2007; Coyne et al., 2008). Hostile sexist men may feel or be perceived as more justified in their use of reactive relational aggression when protecting their social standing than women.

However, it is surprising that gender did not moderate the association between hostile sexism and proactive relational aggression. Reactive relational aggression is generally more related to maladjustment, including difficulty with peers, than proactive relational aggression (Card & Little, 2006). Since women tend to care more about maintaining positive social relationships (Yang & Girgus, 2019), hostile sexist women might be less inclined than hostile sexist men to use reactive aggression, but they might be just as inclined to use proactive relational aggression, since it doesn’t have the same social cost as the reactive function. Nevertheless, since reporting the perpetration of proactive relational aggression was quite rare among the participants, it would be relevant to test the role of gender in the association between hostile sexism and proactive relational aggression in a study using a larger sample since the analyses would be better powered. The lack of variability in the participants’ responses creates little difference to predict in the current study.

### Limitations

First, since the sample was composed of participants who come predominantly from Quebec culture, it would be interesting to look at how sexism might be internalized and affect young adults’ aggressive behaviors in other cultural contexts. The influence of other forms of oppressive beliefs (i. e., racism, homophobia, transphobia) on relational aggression could also be investigated in the future. Second, the cross-sectional nature of this study limits the possibility of drawing conclusions regarding the direction of the associations, which would need to be confirmed by longitudinal studies. Considering that the measures used are primarily self-reported, it would also be relevant to assess whether the results remain the same using a peer-completed measure of relational aggression, for instance. Third, the low reliability indices of the relational aggression scales obtained suggest caution in interpreting the results. Finally, as gender minority status was not included in the analyses, the results do not represent the reality of gender minorities. It would be important to examine gender attitudes and their influence on relational aggression use in a sample with more gender diversity.

Despite these limitations, this study is one of the first to test the associations between hostile and benevolent sexism and relational aggression functions. The use of a large mixed sample including a high proportion of participants with a history of conduct problems maximized the proportion of young adults likely to report aggressive behaviors. The results show the relevance of looking at macro-systemic factors such as sexist attitudes to test their influence on young adults’ aggressive behaviors.

## Conclusion and future recommendations

In conclusion, hostile sexism was associated with the perpetration of reactive and proactive relational aggression, and in the case of reactive aggression, the association was stronger for men that it was for women. Men’s endorsement of this form of sexism is thought to reflect a desire to dominate the gendered hierarchy that is consistent with how relational aggression can be used to control peer relationships. Since this paper is pioneering in the study of the association between sexism and relational aggression, further research would be needed to support the links between these variables. For instance, it would be interesting to examine the distinct influence of both forms of sexism on relational aggression behaviors not only regarding the gender of the aggressor, but also the gender of the victim.

The findings from this study highlight the importance of prevention of sexist attitudes among youth, not only to counter the development of discriminatory biases, but also to improve the way young adults treat their peers. Finally, this study contributes to bridging the gap between research on gender-related attitudes such as sexism and behavioral research. The intersection between these fields of research sheds light on the influence of socialization and gender relations on individuals’ interpersonal behaviors.

## Supplemental material


Supplemental material - Is sexism associated with the use of relational aggression by young adults? The moderating effect of gender
Supplemental material for Is sexism associated with the use of relational aggression by young adults? The moderating effect of gender by Marion Chatelois, Stéphanie Boutin, Alexa Martin-Storey and Michèle Déry, Mélanie Lapalme in Journal of Social and Personal Relationships

## Data Availability

Data available upon request from author Stéphanie Boutin, PhD.*

## Note

1. As the participants were all fluent French speakers, three bilingual members of the longitudinal study’s research team individually translated each item from English to French. They then met to pool their translations and, with the research coordinator, reach a consensus that would be suitable for young adults in Quebec, while still being as representative as possible of the original. The translation was subsequently approved by the principal researcher.
