# Supplementary material for: Is sexism associated with the use of relational aggression by young adults? The moderating effect of gender
Source: J Soc Pers Relat. 2026 Feb 28;43(8):2197–220. doi: 10.1177/02654075261430507 (PMC13283747; doi:10.1177/02654075261430507)
Supplement: Supplemental material - Is sexism associated with the use of relational aggression by young adults? The moderating effect of gender [file sj-pdf-1-spr-10.1177_02654075261430507.pdf]

Supplementary Table 1

Results of the regression model predicting proactive and reactive relational aggression

	Proactive relational aggression				Reactive relational aggression			
	B	*SE*	95% CI	*p*	*B*	*SE*	95% CI	*p*
Gender	-0.02	0.03	[-0.07, 0.04]	.496	-0.05	0.03	[-0.13, 0.04]	.272
Conduct problems	0.02	0.02	[-0.02, 0.07]	.331	0.06	0.04	[-0.01, 0.13]	.100
Hostile sexism	0.06	0.02	[0.02, 0.10]	.003	0.11	0.03	[0.06, 0.16]	.000
Benevolent sexism	-0.00	0.02	[-0.03, 0.03]	.970	0.02	0.02	[-0.03, 0.06]	.522

Note. B = Unstandardized beta; CI = Confidence intervals
